# Discovery of Bile Salt Hydrolase Inhibitors Using an Efficient High-Throughput Screening System

**DOI:** 10.1371/journal.pone.0085344

**Published:** 2014-01-14

**Authors:** Katie Smith, Ximin Zeng, Jun Lin

**Affiliations:** Department of Animal Science, University of Tennessee, Knoxville, Tennessee, United States of America; University of California, Davis, United States of America

## Abstract

The global trend of restricting the use of antibiotic growth promoters (**AGP**) in animal production necessitates the need to develop valid alternatives to maintain productivity and sustainability of food animals. Previous studies suggest inhibition of bile salt hydrolase (**BSH**), an intestinal bacteria-produced enzyme that exerts negative impact on host fat digestion and utilization, is a promising approach to promote animal growth performance. To achieve the long term goal of developing novel alternatives to AGPs, in this study, a rapid and convenient high-throughput screening (**HTS**) system was developed and successfully used for identification of BSH inhibitors. With the aid of a high-purity BSH from a chicken *Lactobacillus salivarius* strain, we optimized various screening conditions (e.g. BSH concentration, reaction buffer pH, incubation temperature and length, substrate type and concentration) and establish a precipitation-based screening approach to identify BSH inhibitors using 96-well or 384-well microplates. A pilot HTS was performed using a small compound library comprised of 2,240 biologically active and structurally diverse compounds. Among the 107 hits, several promising and potent BSH inhibitors (e.g. riboflavin and phenethyl caffeate) were selected and validated by standard BSH activity assay. Interestingly, the HTS also identified a panel of antibiotics as BSH inhibitor; in particular, various tetracycline antibiotics and roxarsone, the widely used AGP, have been demonstrated to display potent inhibitory effect on BSH. Together, this study developed an efficient HTS system and identified several BSH inhibitors with potential as alternatives to AGP. In addition, the findings from this study also suggest a new mode of action of AGP for promoting animal growth.

## Introduction

One of the primary means that food animal producers seek to enhance growth performance is through the use of antibiotic growth promoters (**AGP**). Typically, AGP are defined as subtherapeutic quantities of antibiotics that enhance weight gain and feed conversion ratio [1], [2]. Although this is a long-established technique with benefits to production that are still evident, concern has increased over the last several decades because AGP exert selection pressures for the emergence and persistence of drug-resistant bacteria that threaten food safety and public health [1], [3]. Consequently, groups such as the World Health Organization have strongly urged proactive limitation on AGP use whereas others have banned them outright, as the European Union did in 2006 [1]. Recent suggestions by the Food and Drug Administration also support phasing out antimicrobials used for growth promotion in food animals [4]. Clearly there is an impetus to discontinue AGP use as an agricultural practice, but concerns regarding animal welfare and economic feasibility remain a concern. For this reason, AGP alternatives which could offset such negative impacts must be investigated.

Targeting the mechanism of how AGP exert their growth promoting effects is a central focus when considering what alternative strategy may be an adequate substitute. Although there is no one all-encompassing means by which AGP improve animal performance, the general scientific consensus is that AGP mediate enhanced growth performance by altering intestinal microbiota. Recent studies using poultry and swine have helped us to understand the relationships between AGP supplementation and gastrointestinal bacterial composition [5]–[13]. The results of such studies prove that AGP create bacterial shifts and alter the microbial diversity of the intestine, suggesting that certain populations may be more related to animal growth than others.

Although the definitive gut microbial community required for AGP-mediated optimal growth promotion is still largely unknown, previous studies have shown that the ability of AGP to promote growth is highly correlated with a decrease in activity of bile salt hydrolase (**BSH**) [14]–[16]. BSH is an enzyme produced by commensal bacteria in the intestine whose main function is to convert conjugated bile salts into unconjugated bile salts [17]. Unconjugated bile acids are amphipathic and able to solubilize lipids for micelle formation; however, when the amide bond is hydrolyzed by BSH, the resulting unconjugated form is much less efficient at doing so. Consistent with this finding, independent chicken studies have demonstrated that AGP usage significantly reduced population of *Lactobacillus* species, the major BSH-producers in the chicken intestine; in particular, *L. salivarius*, the dominant lactic acid bacterium present in the chicken intestine, was reduced in response to AGP treatment [5], [7], [16], [17], [18], [19]. Thus, AGP may promote animal growth by reducing the activity of BSH, an enzyme exerting negative impact on host fat digestion and utilization.

Based on these findings, inhibition of BSH activity using specific inhibitors is likely a promising approach to improve growth performance of food animals. This hypothesis is supported by our recent study [20] in which a BSH enzyme with broad substrate specificity from a chicken *L. salivarius* strain [21] was identified and used for evaluating a panel of dietary compounds. In this study [20], discovery of copper and zinc compounds as potent BSH inhibitors offered a potential explanation as to why adding high concentrations of dietary copper and zinc can improve growth performance and feed efficiency of poultry [22]–[25] and swine [26]–[29]. To further test our hypothesis and develop alternatives to AGP, a significant technical hurdle is to identify potent, safe, and cost-effective BSH inhibitors. Modern computational approaches, such as homology modeling and molecular docking, would be helpful for this purpose. However, success of such structure-based computations in the discovery of BSH inhibitor relies on the availability of the defined structures of major BSH enzymes, which is still lacking at present. Since hydrolysis of soluble unconjugated bile salts by BSH generates insoluble unconjugated bile salts that could form significant precipitations [17], we took advantage of this unique hydrolysis feature and developed a high-throughput screening (**HTS**) method to rapidly and efficiently identify BSH inhibitors in this study. Subsequently, a pilot HTS using a diverse compound library identified several promising BSH inhibitors with potential as alternatives to AGP. Notably, the findings from our HTS and quantitative BSH assay also suggest a new mode of action of AGP; specifically, some AGP (e.g. tetracyclines and roxarsone) can directly inhibit intestinal BSH, likely leading to enhanced lipid metabolism and growth performance.

## Materials and Methods

### Purification of Recombinant BSH (rBSH)

The rBSH used in this study was purified from an *E. coli* construct that produces His-tagged BSH with broad substrate specificity from a chicken *L. salivarius* [20]. Expression of the His-tagged rBSH and purification by nickel-nitrilotriacetic acid (Ni^2+^ -NTA) affinity chromatography was carried out as described in our previous publication [20], [30], [31].

### Standard BSH Activity Assay

Determination of BSH activity was performed in microcentrifuge tube by following a two-step procedure to ascertain the amount of liberated amino acids from sodium glycocholate as detailed in our recent publication [20]. All experiments were performed in triplicate. The BSH activity was expressed as 1 µmol of amino acids liberated from the substrate per minute per mg of BSH.

To determine inhibitory effect of a specific compound on the activity of BSH, the rBSH was incubated with or without specific compound for 30 min at 37°C prior to the addition of substrate sodium glycocholate. The standard BSH activity assay was performed to determine the residual activity. A final concentration of specific compound in a BSH activity assay was routinely 5 mM; if needed, specific compound was also serially diluted for dosing experiment. A control without compound added was set up in each independent experiment. All assays were performed in triplicate. Percentage inhibition was calculated by dividing the inhibited activity (mean activity of control – mean residual activity of presence of a compound) relative to the mean activity of control and then multiplied by 100.

### Development of Microplate Precipitation-based BSH Activity Assay

**Figure 1:**
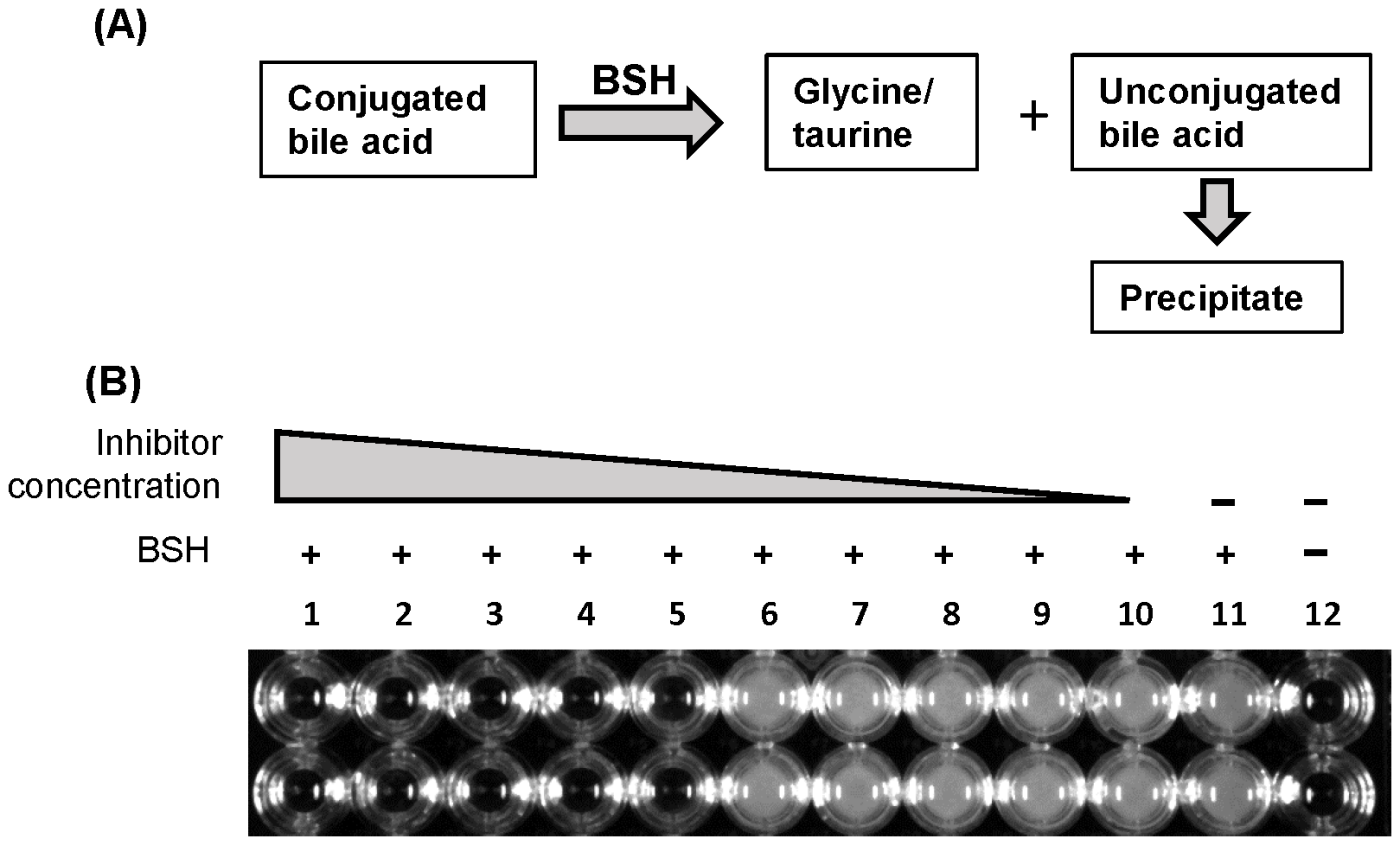
Optimization of conditions for high-throughput screening of BSH inhibitors. (A) Principal of the strategy for HTS screening of BSH inhibitors. (B) Proof of concept experiment to demonstrate the feasibility of the HTS methodology in 96-well plate. Using 200 µl of total reaction volume, BSH inhibitor was added at a final concentration of 0.5 mM in column 1 with 2-fold serial dilution through column 10. KIO_3_ is used as an example in duplicate rows. The purified BSH was added at final concentration of 8 µg/ml in column wells 1–11. Column 12 served as a negative control with no enzyme and BSH inhibitor added.

In order to move towards creating a rapid method of screening for BSH inhibitors, a microtiter plate-based BSH activity assay was developed based on the publication by Tanaka *et al*. [32] with modifications. This is a precipitation-based assay that is based on the fact that hydrolysis of conjugated bile acid substrates will produce deconjugated bile acids which are insoluble at the reaction pH and can easily be visualized as a white precipitate (Fig. 1A).

For initial assays, 10 µl of rBSH (1 µg/µl) was added to the bottom of a clear 96-well microtiter plate with a round bottom. To this, 190 µl of reaction mix (178 µl of reaction buffer [0.1 M sodium-phosphate, pH 6.0], 10 µl of glycocholic acid [100 mM], and 2 µl of 1 M DTT) was added for a total reaction volume of 200 µl. Plates were incubated at 37°C for up to 6 hours. Precipitation of insoluble unconjugated bile salts was monitored every 30 minutes by visual observation concomitant to absorbance measurement at 600 nm (A_600_) using a microplate reader (model Multiskan EX; Thermo Fisher Scientific, Vantaa, Finland). With this general assay procedure, different parameters were altered to determine the optimal assay conditions, which include the stock concentration of the rBSH (from 8 µg/µl to 0.027 µg/µl), pH of reaction buffer (6.0 or 6.5), incubation temperature (20°C, 37°C, or 40°C), conjugated bile salt substrate (glycocholic acid, glycodeoxycholic acid, taucholic acid, and taurodeoxycholic acid), and the final concentration of conjugated bile salt (ranging from 0 mM to 50 mM).

After the assay conditions were optimized for the 200 µl reaction in a 96-well plate, a proof of concept assay was performed to demonstrate the feasibility of this procedure to screen compounds for BSH inhibition by using known BSH inhibitors that were previously determined [20]. The tested inhibitors include KIO_3_, NaIO_4_, NaIO_3_, CuCl_2_, and Na_2_SeO_3_. Before adding the reaction mix, 10 µl of inhibitor was added to the 10 µl of rBSH in the bottom of each well and mixed gently by pipetting. Inhibitors were added at a final concentration of 0.5 mM in column 1 with twofold serial dilutions through column 10 of the plate. As a control, two columns on each plate served as controls: a positive control with BSH and reaction mix only and a negative control with no BSH and inhibitor added. Assays were performed in duplicate.

After all conditions were optimized, including use of inhibitors, the effectiveness and reproducibility of the microplate precipitation-based assay were further evaluated with the entire reaction scaled down to a 50 µl total volume with modification of the volume of specific component to meet the needs of HTS using 384-well microplate. For this purpose, an additional assay was performed using dimethyl sulfoxide (DMSO), the library compound solvent, as a control to rule out possible background noise caused by DMSO.

### High-throughput Screening of BSH Inhibitors

High-throughput screening (**HTS**) of BSH inhibitors was performed in the HTS facility at the Vanderbilt Institute of Chemical Biology (Nashville, TN). The library (2,240 compounds in 7 source plates from Spectrum) includes biologically active and structurally diverse compounds of known drugs, experimental bioactives, and pure natural products. All compounds tested have been dissolved in DMSO at a concentration of 10 mM. Briefly, 0.25 µl of a specific compound from the Spectrum Collection library was transferred into the bottom of a clear 384-well microplate (Greiner cat # 781182) with flat bottom by using the Echo 550/555 Liquid Handlers (Labcyte). Next, 12.5 µl of BSH (0.16 µg/µl prepared with reaction buffer) was added using the Multidrop Combi reagent dispenser (Thermo Scientific) and shaken for 5 minutes. Finally, 37.5 µl of reaction mix (32 µl of reaction buffer [0.1 M sodium-phosphate, pH 6.0], 2.5 µl of taurodeoxycholic acid [200 mM], and 0.5 µl of 1 M DTT) was added using the Multidrop Combi for a total reaction volume of 50 µl. The plates were subsequently shaken for 5 minutes to insure thorough mixing and spun to pull any reaction mixture back into the bottom of the well before incubation. Plates were incubated at 37°C with humidity and 5% CO_2_ and absorbance measured every hour at 600 nm (*A*_600_) for 4 hours using a SpectraMax M5 (Molecular Devices) with temperature controlled at 37°C; visual observations were concomitantly documented for precipitation. The reader on the system is connected via intranet to the facility’s network, enabling automated data acquisition, analysis, visualization, and archival using a powerful management and chemiinformatics tool, Accelrys Pipeline Pilot; all data are stored in an Oracle database. Each plate contained three controls manually added with a multichannel pipette in a predetermined pattern to the side wells. Control 1 consisted of BSH, DMSO solvent, and reaction mix; control 2 contained a known inhibitor, BSH, and reaction mix; control 3 contained DMSO and reaction mix only.

Prior to screening the compound library, the above HTS protocol was validated twice in a full 384-well plate with checkerboard design containing activity controls (BSH, reaction mix containing substrate, and solvent DMSO) and inhibition controls (BSH, reaction mix containing substrate, and NaIO_3_).

### Selection and Validation of Identified BSH Inhibitors

A preliminary list of BSH inhibitor hits was generated after the above HTS; the wells that were completely clear after 4 hours of incubation are deemed as hits. Extensive review of relevant material safety data sheet and literature were performed for the hits with emphasis on availability, stability, toxicity, cost, and environmental impact.

The selected BSH inhibitor candidates were subjected to further *in vitro* validation using the 96-well microplate assay as well as the standard 2-step BSH assay as described above. The standard BSH assay is essential to confirm if the identified compounds are indeed real BSH inhibitors because we cannot completely rule out a possibility that certain compounds may cause less or no precipitation in reaction mix in a BSH activity-independent manner. The stock solutions of selected BSH inhibitors were purchased directly from the HTS facility at the Vanderbilt Institute of Chemical Biology, which had been solubilized in DMSO; compounds were stored at −20°C after arrival. If needed, dosing experiment was further performed to evaluate the potency of specific inhibitor.

Although the hit compounds belonging to an antibiotic family were not considered as potential alternatives to AGP, the following antibiotics were also chosen from the hit list and subjected to standard BSH assay to confirm if they had a direct inhibitory effect on BSH activity: cloxacillin sodium, cephradine, oxytetracycline, demeclocycline hydrochloride, and methacycline hydrochloride. In addition, following antibiotics that have been used as AGP were selected for evaluating their inhibitory effect on BSH activity using the standard 2-step BSH assay: doxycycline hydrochloride, roxarsone, tylosin, bacitracin, erythromycin, lincomycin, streptomycin, and sulfamethazine.

## Results

### Optimal Conditions for Microplate Precipitation-based BSH Activity Assay

For initial assay in 96-well plate with final volume of 200 µl, rBSH concentration, reaction buffer pH, incubation temperature and length, substrate type and concentration were altered in separate assays for assessment. As expected, a pH of 6.0 and an incubation temperature of 37°C, both of which are used in the standard 2-step assay, were conducive to efficient BSH hydrolysis. An incubation length of 2 to 4 hours was targeted in order to get several progressive measurements during HTS to monitor the dynamics of precipitation development. Given that using large quantities of rBSH could cause the reaction to proceed too quickly, rBSH was serially diluted for the plate assay; it was determined that using 10 µl of rBSH at the concentration approximately 0.16 µg/µl elicited adequate hydrolysis of substrate with maximal precipitation occurring after 2 hours of incubation. Regarding the choice of substrate, taurodeoxycholic acid was better than other tested conjugated bile salts because it elicited most observable BSH activity response (generation of white precipitation), which was also able to be corroborated by measurements using a microplate reader (data not shown).

With the above parameters optimized, a proof-of-concept experiment using known BSH inhibitors was performed. As shown in Fig. 1B, in the presence of high levels of a specific BSH inhibitor (KIO_3_), the activity of BSH was inhibited, which was reflected by displaying clear, transparent wells (columns 1–5). In the presence of low concentrations of the BSH inhibitor (columns 6–10) or in the absence of the BSH inhibitor (columns 11, Fig. 1B), the reaction mix became turbid due to the precipitation of unconjugated bile salts resulting from active BSH enzyme. Other selected BSH inhibitors also displayed similar pattern as shown in Fig. 1B (data not shown), demonstrating the feasibility of the optimized microplate precipitation-based methodology for discovery of BSH inhibitors using HTS.

When the assay was further scaled down to a 50 µl total reaction volume, a prolonged incubation length (3–4 hours) was needed for precipitation to fully develop. DMSO, the solvent used for HTS library compounds, had little effect on precipitation formation with final concentration as high as 50 mM (data not shown).

### HTS Discovery of BSH Inhibitors

At the HTS facility at the Vanderbilt Institute of Chemical Biology, all assays were performed in 384-well plate with 50 µl total reaction volume. Prior to screening of the compound libraries, validation assays containing different controls were carried out to ensure the system was optimal for actual inhibitor screening and that the optimized conditions translated well to this HTS format. Notably, to ensure consistent and accurate delivery of rBSH into 384-well plate using dispenser, the optimized 50-ul reaction mix setting was further modified by using larger volume of diluted rBSH (12.5 µl rather than 2.5 µl) despite final concentration of rBSH in reaction mix unchanged. With this minor on-site modification, the results of validation assays indicated that the assay could clearly distinguish between wells with or without precipitation as a result of BSH hydrolysis. Both negative controls (no BSH) and those using BSH inhibitor (NaIO_4_ or KIO_3_) remained clear in stark contrast to positive controls (no inhibitor) that displayed obvious precipitation; the visual results were also consistent with spectrometer reading (data not shown).

**Figure 2:**
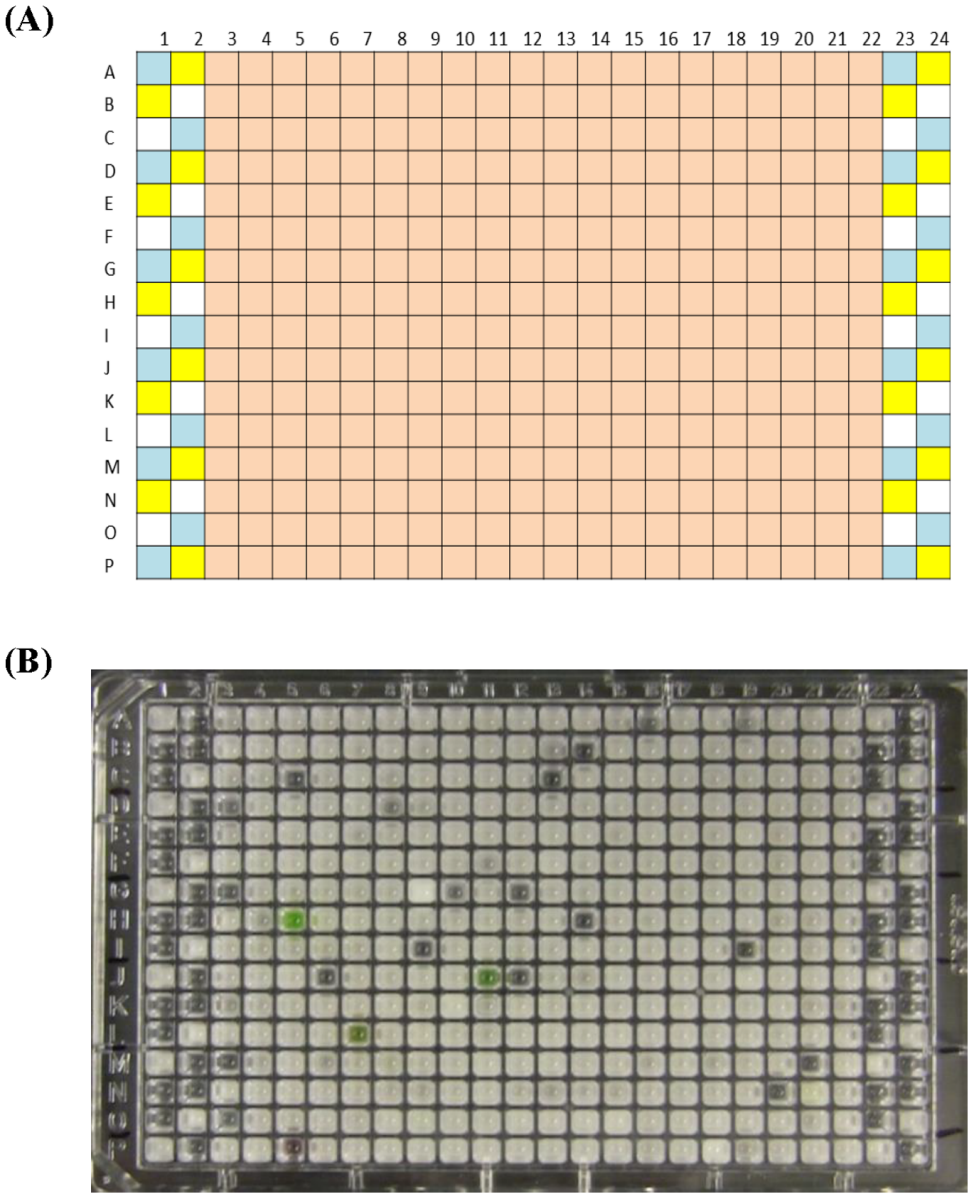
Representative result of high-throughput screening of BSH inhibitors. (A) Plate layout for screening BSH inhibitors. Pink boxes (columns 3–22) indicate test wells that contain library compounds of interest, BSH, and reaction mix containing substrate. Library compounds were shot into the well bottom using Echo 550/555 and enzyme and reaction mix were added using Multidrop Combi. Controls were added manually to the side wells (columns 1–2 and 23–24): blue boxes indicate activity controls (BSH, reaction mix containing substrate, and solvent DMSO), yellow boxes correspond to inhibition controls (BSH, reaction mix containing substrate, and NaIO_3_), and white boxes are negative controls with no BSH added but include reaction mix as well as substrate. (B) The HTS results represented by one 384-well plate. Control wells indicate the assay proceeded normally. The wells in columns 3–22 that appeared clear, regardless of alternative color due to compound, and had low absorbance readings were considered hits (putative BSH inhibitors).

With the successful validation assays using HTS system, a pilot HTS using a 2,240-compound library was performed; plate setting was shown in Fig. 2A. A total of 7 plates were used for this screening, which led to 107 hits that were considered potent inhibitors. Hits were selected based on visual observation (the clear wells from lane 3 to lane 22 in a representative plate as shown in Fig. 2B) and corroborated via spectrophotometric measurement.

### Validation of Selected HTS Hits with Potential as Feed Additives

Preliminary review of biochemical information of the corresponding hits eliminated most of compounds and identified 10 compounds that potentially could be used as feed additives for animal industry (Table 1). These 10 compounds were subjected to further validation using the plate assay as well as the 2-step activity assay to ascertain the quantitative inhibitory effect. The standard 2-step BSH activity assays identified two compounds as false positive (chrysophanol and folic acid); these two compounds also failed to inhibit BSH using 96-well plate assay.

**Table 1:** Effect of selected HTS hits on BSH activity^a^.

Compound	Formula (MW)	% Inhibition
Caffeic Acid PhenethylEster (CAPE)	C_17_H_16_O_4_ (284.31)	99.7
Riboflavin^b^	C_17_H_20_N_4_O_6_ (376.37)	99.3
Epicatechin monogallate	C_20_H_28_O_4_ (332.44)	98.6
Gossypetin	C_15_H_10_O_8_ (318.24)	97.3
Carnosic Acid	C_20_H_28_O_4_ (332.44)	96.8
Menadione	C_11_H_8_O_2_ (172.18)	87.2
Purpurogallin	C_11_H_8_O_5_ (220.18)	83.0
Theaflavanin	C_20_H_16_O_8_ (384.34)	18.1
Chrysophanol^c^	C_15_H_10_O_4_ (254.24)	3.7
Folic Acid^b^	C_19_H_19_N_7_O_6_ (441.40)	−10.8

^a^ Unless specified, the final concentration of compound in the reaction mix was 5 mM to achieve optimal resolution with the quantitative BSH activity assay.

^b^ The final concentration of riboflavin in reaction mix was 1 mM.

^c^ The final concentration of chrysophanol in reaction mix was 1.25 mM.

^d^ The final concentration of folic acid in reaction mix was 1.5 mM.

**Figure 3:**
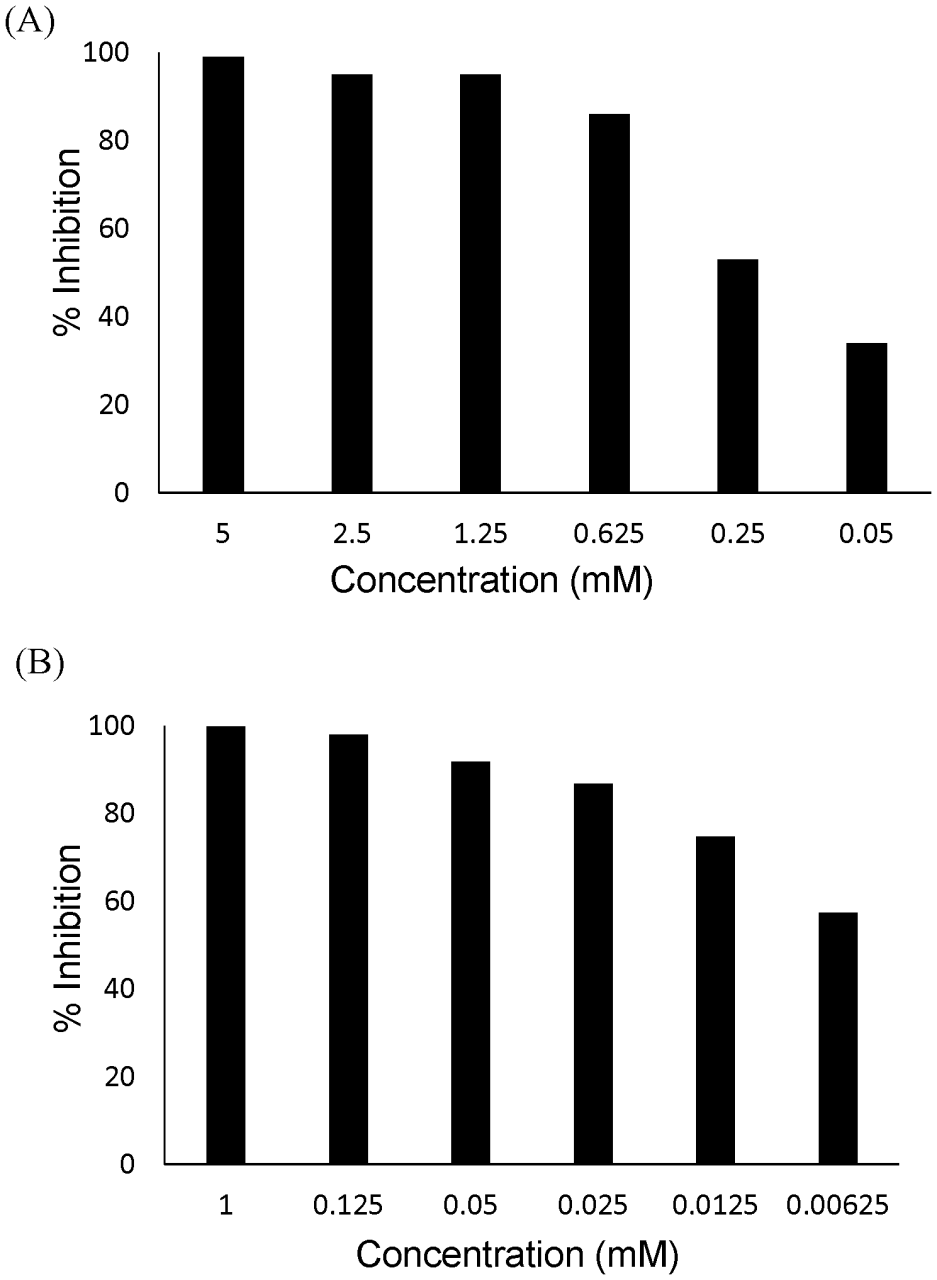
Dosing effects of selected BSH inhibitors on BSH activity. (A) Inhibition of BSH activity by caffeic acid phenethyl ester (CAPE). (B) Inhibition of BSH activity by riboflavin. All assays were performed in triplicate. The procedure is detailed in Materials and Methods.

Majority of the selected hits displayed potent inhibitory effect on BSH (Table 1). Notably, caffeic acid phenethyl ester (CAPE), riboflavin, epicatechin monogallate, gossypetin, and carnosic acid consistently inhibited activity by over 95% in independent experiments (data not shown). In particular, despite its relative lower final concentration (1 mM) in reaction mix due to solubility issue, riboflavin still exerted strong inhibition on BSH activity (>99% of inhibition) (Table 1). Because we are particularly interested in CAPE and riboflavin as AGP alternatives, dosing experiments were conducted to examine if they could inhibit BSH activity at lower concentrations. CAPE still inhibited rBSH activity by more than 50% at a final concentration of 0.25 mM (Fig. 3A) and riboflavin by more than 50% at a final concentration as low as 0.00625 mM (Fig. 3B).

### Direct Inhibitory Effect of Antibiotics on BSH

**Figure 4:**
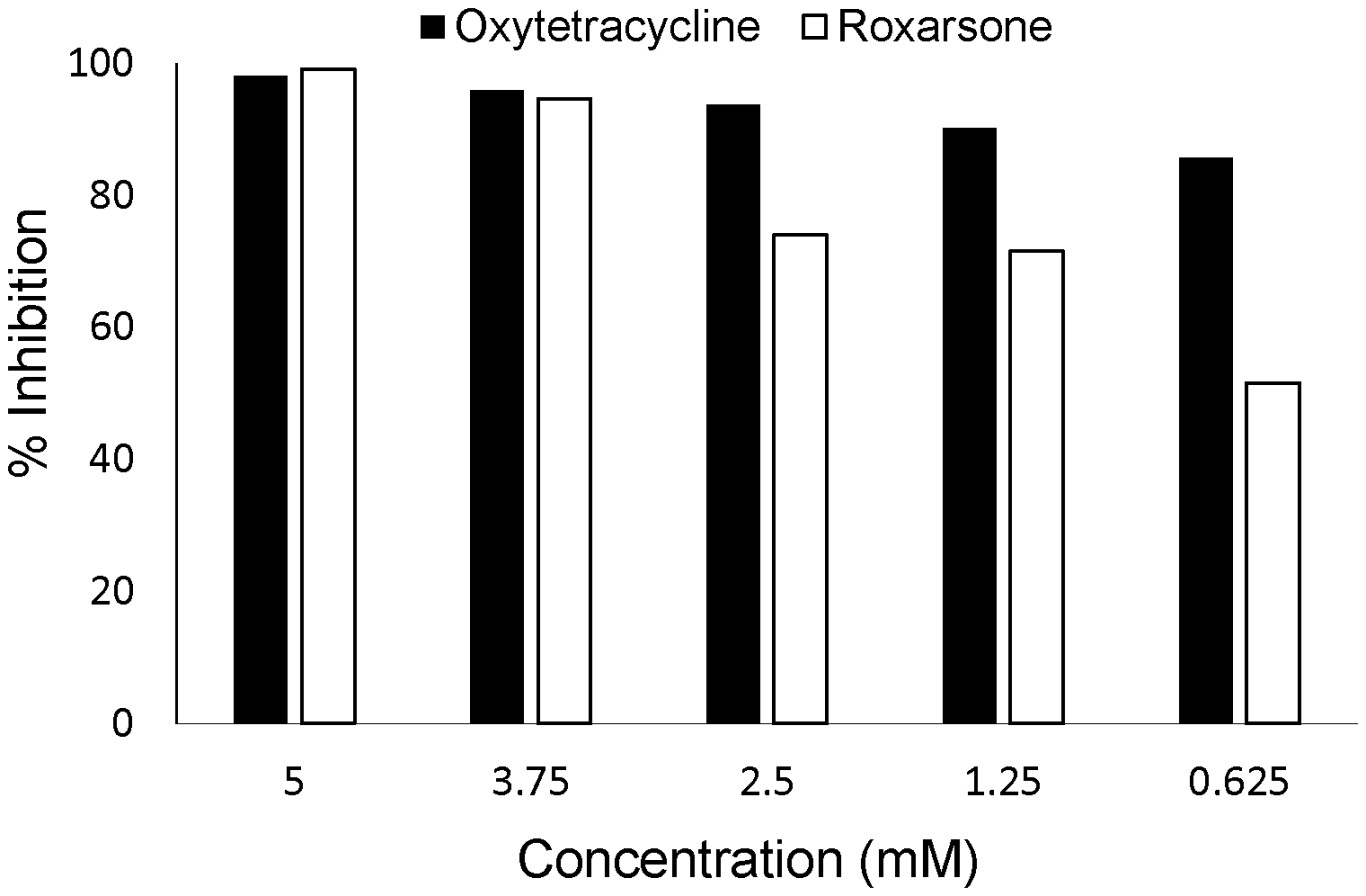
Dosing effects of oxytetracycline and roxarsone on BSH activity. Black bars represent Inhibition of BSH activity by oxytetracyline and white bars by roxarsone. All assays were performed in triplicate. The procedure is detailed in Materials and Methods.

Interestingly, a group of antibiotics were also HTS hits, including tetracycline and β-lactam antibiotics that have been widely used as AGP. This finding prompted us to speculate that direct inhibition of BSH activity by some antibiotics may be a new mode of action of AGP. To partly test this, we further examined a panel of antibiotics that have been used as AGP in food animals and determined if different classes of antibiotic make a difference in inhibition potential. As shown in Table 2, tetracycline antibiotics were consistently potent inhibitors. Additionally, roxarsone, an arsenical drug that has been extensively used in poultry production, also displayed high inhibitory effect on BSH activity. Both roxarsone and oxytetracycline inhibited BSH activity by over 95% when tested at a final concentration of 5 mM (Table 2). In an additional dosing experiment, oxytetracycline and roxarsone still inhibited enzyme activity by approximately 86% and 52%, respectively, at a final concentration of 0.625 mM (Fig. 4). Other tested antibiotics, such as β-lactam and lincosamide, displayed strong but relatively lower inhibitory effect on BSH activity (Table 2). However, the macrolide and bacitracin antibiotics had weak or no inhibitory effect on BSH.

**Table 2:** Effect of different classes of antibiotics on BSH activity.

Antibiotic class	Antibiotic name	% Inhibition
Tetracycline	Oxytetracycline*	97.4
	Demeclocycline Hydrochloride*	92.0
	Methacycline Hydrochloride*	90.2
	Doxycycline Hydrochloride	92.9
β-lactam	Cloxacillin*	61.2
	Cephradine*	61.8
Arsenicals	Roxarsone	98.4
Lincosamide	Lincomycin	67.3
Sulfonamide	Sulfamethazine	48.7
Macrolide	Tylosin	23.8
	Erythromycin	36.5
Peptides	Bacitracin	−17.8

The antibiotic hits by HTS.

## Discussion

Antibiotic growth promoters (**AGP**) undoubtedly have a positive influence on animal production in terms of the improvements they confer on feed utilization efficiency, animal welfare as well as the resulting financial gains. However, antibiotic resistance of zoonotic origin threatens the supply of food animals and public safety and, thus, is a compelling reason to discontinue their inclusion as feed additives. Likewise, elimination of dietary AGP will necessitate alternative strategies to compensate for setbacks to production, as have occurred in some areas where bans have been instituted [33]. Consequently, different products have been proposed as alternatives for AGP, including essential oils, prebiotics, probiotics, exogenous enzymes, and organic acids, among others, to change gut microbiota for enhanced animal health and growth performance [34]. However, very limited data is available to justify the choice of specific bacterial species or products for such microbiota manipulation despite the link between AGP induced growth promotion and altered gastrointestinal microflora [1], [2].

A promising strategy for developing AGP alternative is targeting general metabolic pathway that is influenced by AGP usage. Specifically, the activity of microbial bile salt hydrolase (**BSH**) in the intestine is a valid object for manipulation since studies repeatedly have observed that AGP use is associated with reduced BSH activity and improved animal growth performance [14]–[16]. Conjugated bile acids play a critical role in lipid solubilization and micelle formation and are the target substrates of BSH [2], [17]. However, deconjugation of bile acids by BSH compromises lipid metabolism and results in attenuated energy harvest. Therefore, we have proposed to develop BSH inhibitor-based alternatives to AGP for enhanced animal production and sustainability [20]; this recent study established a solid platform for us to discover novel BSH inhibitors.

In this study, we improved the capacity to screen large quantities of compounds in a time efficient manner by developing and validating a HTS assay to rapidly identify BSH inhibitors. Overall, the ability of the HTS system to identify BSH inhibitors proved to be a reliable means of inhibitor detection. Validation work was able to confirm that many of our chosen HTS hit compounds displayed high levels of BSH inhibition as expected. However, false positives were also present; chrysophanol and folic acid, both recognized as inhibitors in the library screen, exhibited minimal enzyme inhibition or even slight enhancement (Table 1), which is likely due to solution stability or chemical properties [35]. For this pilot screening, we only had the time and resources to do one all-encompassing screen. A second re-screen, including only a subset of compounds of interest, would have been beneficial in confirming our suspected compounds as legitimate inhibitors. Thus, in future HTS using a much larger compound library, it is essential to select all positive hits individually from corresponding source plates – a so-called ‘cherry picking’ procedure – for confirmation using the same protocol.

The Spectrum Collection library that was screened contained a wide range of structurally diverse compounds, including drug components, natural products, and other bioactive constituents. Several potent inhibitors confirmed by validation assay possess interesting beneficial qualities. Carnosic acid and epicatechin monogallate are both nutraceuticals with noted antioxidant and neuroprotective effects [36]–[40]. Additionally, carnosic acid is an anti-inflammatory [41] and epicatechin monogallate displays some chemopreventive [42] and antimicrobial properties [43]. Similarly, gossypetin was among the powerful inhibitors and also is noted to be both anti-inflammatory [44] and an antioxidant [45]. Each of these compounds warrants further animal research, and should availability permit, growth performance studies could reveal more as to their potential use. However, two particular compounds stood out as potent inhibitors with definite high potential as novel alternative to AGP: riboflavin and caffeic acid phenethyl ester (CAPE, also called ‘phenethyl caffeate’). Riboflavin, or vitamin B2, plays a key role in energy metabolism and is a conenzyme in numerous redox reactions [46]. Aside from contributions to animal physiology, it is water-soluble and causes no known toxicities from supplementation at upper limits [47], although even at the lowest concentrations tested, riboflavin was still an extremely potent BSH inhibitor (Fig. 3B). Because it is an FDA-approved feed additive with well-established metabolic function and would be readily available to incorporate into feed, riboflavin likely may be an acceptable candidate to improve growth performance for food animals. CAPE, on the other hand, is an emerging bioactive studied because it has antioxidant [48]–[50], anticarcinogenic [51], [52], anti-inflammatory [50], [53], [54], immunomodulatory [52], and antimicrobial [54] effects. It is a phenolic component of propolis and can be directly extracted or artificially synthesized by several methods [55]. Although much research has been done to characterize the aforementioned effects of CAPE, no published data exist concerning its effects on growth performance of food animals. Because CAPE can inhibit BSH at low concentrations, a trial of this natural product as a feed supplement is also highly warranted.

For initial evaluation of HTS hits, we eliminated the hits that fall in antimicrobial category when selecting the BSH inhibitors with potential as alternative to AGP. However, the finding that some antibiotic hits, such as tetracyclines that have been widely used as AGP, called our attention to a possible new mechanism of AGP: AGP may have a direct effect on the function of gut BSH enzymes [17], [56] aside from modulating gut microbial ecology for enhanced growth performance. Using standard BSH assay, in this study, we have confirmed that the tested tetracycline and β-lactam antibiotics could directly inhibit BSH activity. Notably, roxarsone, an arsenical drug that has been used in the poultry industry to improve weight gain, feed conversion, and pigmentation as well as to control coccidiosis [57], [58], also displayed potent inhibitory effect on BSH. Currently little is known about how roxarsone is metabolized by chickens to mediate growth promotion [58] although it is suspected it may be due to its angiogenic potential [59]. Our results suggest that roxarsone as well as some other AGP (e.g. tetracycline) may also exert their growth-promoting effect on food animals by direct inhibition of intestinal BSH enzymes for enhanced lipid metabolism and energy harvest. Thus, this unexpected finding suggests a new mode of action of AGP for promoting animal growth.

The pilot HTS in this study confirmed that our HTS protocol is a valid BSH inhibitor detection method. Future screens using larger compound libraries may reveal more novel BSH inhibitors. Having a greater number of applicable compounds will allow us to choose those with the most redeemable qualities that would include animal and environmental safety, cost, and availability. In addition, large animal trials are needed to determine the effect of ‘champion’ BSH inhibitors on growth performance of food animals. While *in vitro* studies are absolutely essential to justify potential AGP alternatives, intestinal *in vivo* conditions are certainly more complex. The *in vivo* stability and bioavailability must be addressed in order to establish the validity and practicality of utilizing such inhibitors as a legitimate alternative to AGP. Moreover, growth performance parameters like body weight gain, feed intake, and feed conversion ratio will need to be measured as well as morphological characteristics of the intestinal tract and meat and carcass quality. This will help to rule out or identify any negative physiological consequences associated with prolonged use of a particular BSH inhibitor. For example, because the BSH inhibitors may improve lipid metabolism, it is important to determine that energy harvest and weight gain is partitioned adequately and not skewed toward excess fat deposition, which would be undesirable for industry and consumers. Thus, comprehensive animal trials are needed to address the above issues and corroborate the performance-boosting benefits of novel BSH inhibitors.

Discovery of BSH inhibitors may help us develop ‘negative-traits-mitigation’ strategy to optimize probiotic products for enhanced growth performance of food animals and profitability of feed additive industry. It is no doubt that dietary probiotics, the normal commensal microorganisms, could exert various beneficial effects on food animals. However, probiotics do impose a variety of potential costs (or detrimental effects) to the animal host as well, which include the production of toxic metabolites, decreased fat digestability due to production of BSH, and the increase of mucus secretion and gut epithelial cell turn-over [2]. Since lactobacilli are dominant BSH-producers in the small intestine, dietary lactobacilli treatment may negatively affect lipid metabolism and energy harvest, consequently imposing negative impact on body weight gain. Recently, two different groups have reported that probiotic supplementation to diets significantly reduced body weight gain, fat digestibility, and feed conversion in broilers [60], [61]. Based on these findings, the investigators have proposed that the detrimental effects of the probiotics on chicken growth are likely attributed to the production of intestinal BSH by lactobacilli probiotics. These recent studies provided physiological evidence for relationship between lipid metabolism and the BSH activity/BSH-producers in the chicken intestine. Therefore, the overall beneficial effects associated with specific probiotics should be carefully evaluated. The BSH inhibitors may be used together with certain probiotics to maximize the beneficial effect of the probiotics by mitigating potential negative impact of probiotics on host fat digestion due to the production of BSH enzyme.

The efficient HTS technology developed in this study should have the broad appeal to the community of researchers who study BSH and develop BSH-based practical applications. It has been increasingly recognized that intestinal BSH plays an important role in host metabolism and energy harvest [17], [56]. The BSH activity has significant impact on host physiology by disturbing conjugated bile salts-mediated fat metabolism and endocrine functions, consequently affecting body weight gain and even cholesterol level in humans [17], [56]. Despite the significance of gut BSH, research on BSH is still in its infancy [62]. Not surprisingly, little information exists concerning the compounds that inhibit or enhance the activity of BSH. Notably, the HTS method described in this manuscript could be easily modified to make it suitable for the discovery of BSH enhancers. Specifically, during our optimization experiments, we have observed that changing some parameters, such as using less rBSH, would prolong incubation time for full precipitation development. With the control of desired reaction time (e.g. 8 hr), the dynamics and magnitude of precipitation formation in each well (reflected by A_600_ value) could be monitored and used for screening BSH enhancers. Since enhanced BSH activity in the intestine could lower cholesterol level in humans [17], [56], we speculate that BSH enhancers have a potential application in human health. Together, this study successfully developed a timely HTS technology and established a solid platform for future large-scale discovery of BSH inhibitors as well as BSH enhancers.
